# Isolated splenic tuberculosis in an immunocompetent child: A case report

**DOI:** 10.1016/j.ijscr.2025.110906

**Published:** 2025-01-20

**Authors:** Saad Andaloussi, Saad Annattah, Mohammed Eljiar, Omar Dalero, Zakarya Alami Hassani, Aziz Elmadi

**Affiliations:** a Department of Pediatric Surgery, Mohamed VI University Hospital, Faculty of Medicine and Pharmacy, Abdelmalek Essaâdi University, Tetouan, Morocco; b Department of Anatomical Pathology, Mohamed VI University Hospital, Faculty of Medicine and Pharmacy, Abdelmalek Essaâdi University, Tetouan, Morocco

**Keywords:** Tuberculosis, Infection, Spleen, Splenectomy, Child, Case report

## Abstract

**Introduction:**

Tuberculosis remains a major public health problem in developing countries. Isolated splenic tuberculosis is a rare form of extrapulmonary tuberculosis, with few cases documented in the literature, occurring mainly in immunocompromised individuals. The purpose of this article is to contribute to the medical knowledge regarding this rare disease and to highlight the diagnostic challenges and management approach.

**Case Presentation:**

A 6-year-old immunocompetent child was admitted with febrile splenomegaly. Initial diagnostic evaluations, including imaging, suggested a possible diagnosis of lymphoma, prompting a laparoscopic splenectomy for diagnostic and therapeutic purposes. Histopathological examination of the splenic tissue revealed features consistent with tuberculosis, despite the absence of a detectable primary focus in the lungs or other organs. Postoperatively, the patient underwent a 6-month course of anti-tubercular therapy, with no recurrence observed during follow-up.

**Discussion:**

Isolated splenic tuberculosis is an uncommon entity, particularly in immunocompetent individuals. The diagnosis is often challenging and delayed due to its nonspecific presentation, which can often mimic other conditions such as lymphoproliferative disorders, metastatic diseases, or other granulomatous diseases. Definitive diagnosis is based on histopathological analysis.

**Conclusion:**

Splenic tuberculosis, though rare in immunocompetent child, should be included in the differential diagnosis list of febrile splenomegaly, particularly in areas where tuberculosis is endemic. Early recognition and appropriate treatment are crucial to prevent complications and ensure favorable outcomes.

## Highlights

• Isolated splenic tuberculosis is a rare form of extrapulmonary tuberculosis, with few cases documented in the literature particularly in immunocompetent children.
• The diagnosis is often challenging and delayed due to its nonspecific clinical and radiological presentation.
• Definitive diagnosis is based on histopathological analysis.
• Early recognition and appropriate treatment are crucial to prevent complications and ensure favorable outcomes.

## 1 Introduction

Tuberculosis remains a major public health problem in developing countries. It can affect any organ and can present with a multitude of clinical manifestations. Extrapulmonary tuberculosis accounts for 15 to 20 % of all cases, while abdominal tuberculosis represents only 3 %, primarily affecting the ileocecal region, mesenteric lymph nodes, and the peritoneum [1]. Splenic involvement in tuberculosis mainly occurs in the context of miliary tuberculosis in immunocompromised patients. Isolated splenic tuberculosis is extremely rare, with only a few cases reported in the literature, especially in immunocompetent children even in countries with a high prevalence of tuberculosis. The diagnosis is difficult and often delayed due to nonspecific clinical and imaging features. We present the case of a six-year-old immunocompetent child with isolated splenic tuberculosis, which was initially misdiagnosed as lymphoma due to its unusual and localized splenic involvement.

Our work has been reported in line with the SCARE criteria [2].

## 2 Presentation of case

**Fig. 1:**
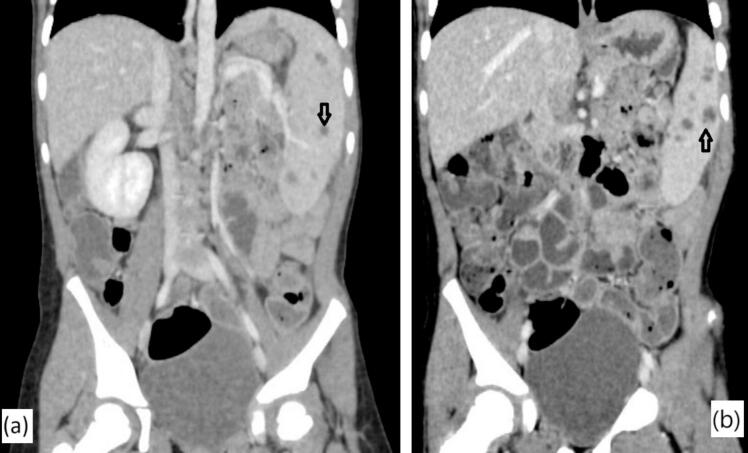
a, b: Contrast-enhanced computed tomography of the abdomen (Coronal Sections) showing mild splenomegaly with multiple hypodense splenic lesions (black arrow).

**Fig. 2:**
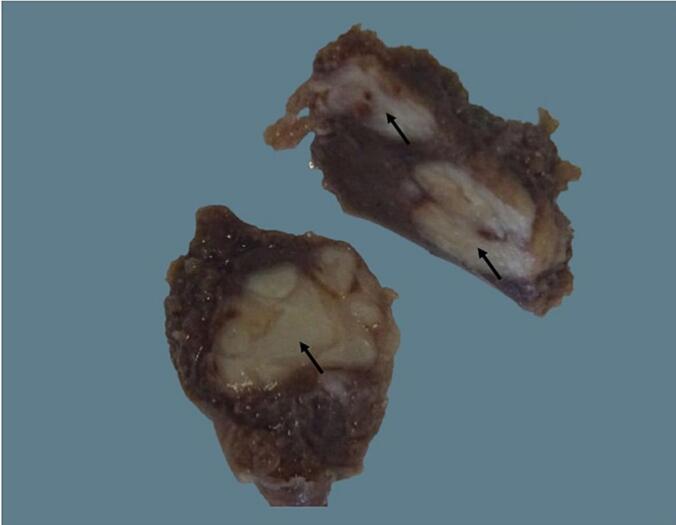
Cut section of resected splenectomy specimen showing whitish foci of caseous necrosis (black arrows).

**Fig. 3:**
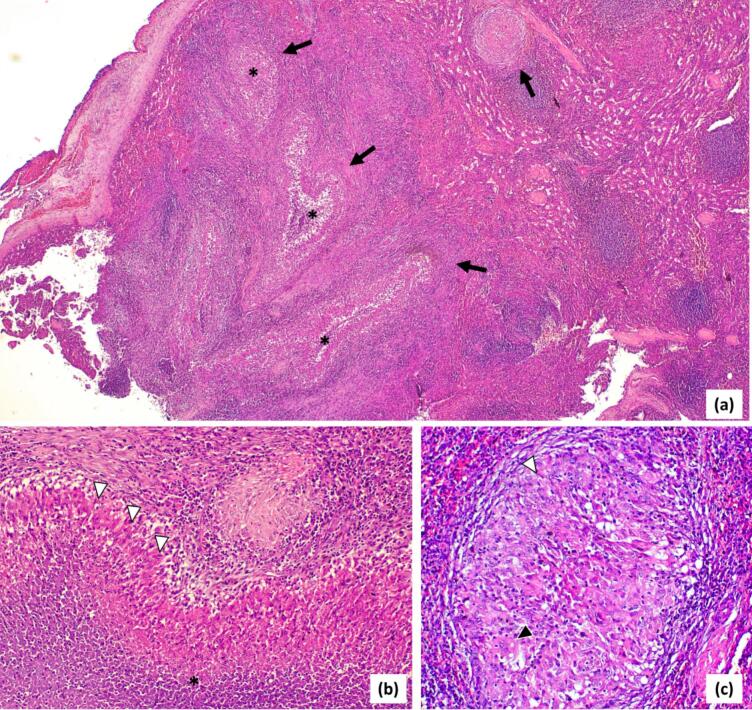
Hemotoxylin and eosin stained section of the spleen showing a significant parenchymal enlargement characterized by a granulomatous inflammatory reaction. The granulomas varied in sizes (black arrow). They were bordered by epithelioid cells (white arrowhead) and multinucleated giant cells (black arrowhead), with central areas of suppurative caseous necrosis (asterisk). Magnification: (a) x20, (b) x200, (c) x400.

A 6-year-old child, with no personal or family history of tuberculosis, presented with pain in the left hypochondrium, intermittent vomiting, and persistent low-grade fever for three months, without chills or night sweats. He was up to date with all his vaccinations, including the Bacillus Calmette-Guérin (BCG) vaccine. On admission, he was 118 cm tall, weighed 20 kg and showed no signs of acute distress. Physical examination revealed a pale but cooperative child with a body temperature of 38.6 °C. His abdomen was mildly tender with a palpable spleen extending 4 cm below the left costal margin. No significant lymphadenopathy was found. Initial complete blood count revealed pancytopenia, characterized by anemia (hemoglobin at 8.2 g/dL), leukopenia (3700 white blood cells/ mm^3^), and thrombocytopenia (119,000 platelets/ mm^3^). Hypersplenism was confirmed on bone marrow aspirate that showed increased cellularity of all cell lines with erythroid hyperplasia and a few megaloblasts. Smear and culture of sputum for acidfast bacilli were negative. Liver and kidney function tests were within normal limits, while the erythrocyte sedimentation rate was 54 mm/h. Serological tests for human immunodeficiency virus (HIV) were negative. The Mantoux test showed an induration of 6 mm, presumed to be a false positive due to the BCG vaccination. However, the Xpert MTB/RIF (*Mycobacterium tuberculosis*/rifampicin) assay was not performed. Chest X-ray showed no abnormalities. Abdominal ultrasonography revealed an enlarged spleen with hypoechoic cystic formations and some splenic hilum lymphadenopathy. Contrast-enhanced computed tomography of the abdomen showed heterogeneous splenomegaly containing multiple hypodense lesions associated with 13 mm peri-hilar lymphadenopathy, primarily suggesting lymphoma (Fig. 1). A splenic biopsy was not performed due to a potential risk of rupture. In view of persistent pancytopenia and splenomegaly, the patient underwent laparoscopic splenectomy for both diagnostic and therapeutic purposes after being vaccinated against *Haemophilus influenzae* and pneumococcus. Macroscopically, the splenectomy specimen contained whitish foci of caseous necrosis (Fig. 2). Histopathological examination revealed a significant parenchymal enlargement characterized by a granulomatous inflammatory reaction. The granulomas varied in sizes. They were bordered by epithelioid cells and Langhan's multinucleated giant cells, with central areas of suppurative caseous necrosis compatible with tuberculosis (Fig. 3). A special preparation with Ziehl-Neelsen stain identified multiple acid-fast bacilli consistent with *Mycobacterium tuberculosis*. Following surgery, oral antitubercular treatment with four drugs was started (isoniazid 10 mg/Kg/day; rifampicin 15 mg/Kg/day; pyrazinamide 35 mg/Kg/day; ethambutol 20 mg/Kg/day) for two months and continued with two drugs (isoniazid 10 mg/Kg/day; rifampicin 15 mg/Kg/day) for an additional four months. At 1-year follow-up, the patient is doing well and gaining weight. Control blood count and ultrasound examinations performed at 1, 6 and 12 months show no recurrence.

## 3 Discussion

Splenic tuberculosis was first described in the literature by Coley in 1846 [3]. This uncommon form of tuberculosis primarily occurs in the context of miliary tuberculosis in immunocompromised patients [4]. Isolated splenic tuberculosis is exceedingly rare, with few cases reported in the literature, especially among immunocompetent children. The patient had no history of tuberculosis and showed no signs of tuberculosis in other organs. Additionally, no immunosuppressive conditions that could have predisposed to this infection were identified.

From a pathomorphological perspective, splenic tuberculosis is categorized into five types: miliary tuberculosis, nodular tuberculosis, tuberculous splenic abscess, calcifying tuberculosis, and mixed-type tuberculosis [5]. Diagnosing splenic tuberculosis is challenging and often delayed due to its nonspecific clinical presentations, which range from asymptomatic cases to various symptoms including fever, weakness, weight loss, abdominal pain, and splenomegaly. In some cases, patients may present with only a prolonged fever of unknown origin [6]. In our case, the clinical presentation consisted of abdominal discomfort accompanied by intermittent vomiting and moderate fever.

Moreover, the reliability of common imaging modalities, including ultrasound and contrast-enhanced computed tomography, in differentiating such lesions from conditions like metastases, lymphoproliferative disorders, and other granulomatous diseases is limited. The rate of misdiagnosis is high, particularly in the absence of a history of tuberculosis in other organs. Ultrasound features of splenic tuberculosis may present as well-defined or poorly defined hypoechoic lesions, with or without calcifications. Computed tomography imaging may reveal hypodense micronodular or macronodular lesions, often associated with heterogeneous enhancement; however, homogeneous splenomegaly can also be observed [7].

Patients with splenic tuberculosis may also exhibit hematological abnormalities. Cytopenia is common, although cases of polycythemia have been reported. An elevated erythrocyte sedimentation rate (ESR) can serve as a diagnostic marker [5,8]. The Mantoux test is often positive; however, in tuberculosis-endemic regions, this finding has limited diagnostic value. When splenic tuberculosis is suspected, fine-needle aspiration, splenic biopsy, or splenectomy is essential for a definitive diagnosis. Histopathological examination reveals characteristic granulomas composed of central caseous necrosis surrounded by epithelioid cells, Langhans giant cells and lymphocytes [9].

Early diagnosis is crucial, as untreated splenic tuberculosis can have a high mortality rate. Anti-tubercular therapy remains the first-line treatment, comprising a two-month intensive phase with isoniazid, rifampicin, ethambutol, and pyrazinamide, followed by a four-month continuation phase with isoniazid and rifampicin. Splenectomy is generally not required as a treatment modality except in specific situations, including failure of medical management, multiple splenic abscesses, or when the diagnosis is uncertain. Laparoscopy serves as a minimally invasive approach for managing this rare condition.

In our case, a diagnosis of lymphoma was initially suspected based on clinical and radiological findings, which led to the decision to perform a laparoscopic splenectomy for both diagnostic evaluation and therapeutic management of persistent hypersplenism. This underscores the necessity for clinicians to maintain a high index of suspicion for tuberculosis in cases of unexplained splenic lesions, even in immunocompetent individuals.

## 4 Conclusion

Isolated splenic tuberculosis, although rare, should be considered in the differential diagnosis of febrile splenomegaly, particularly in regions where tuberculosis is endemic regardless of the patient's immunological status. The nonspecific clinical presentation and overlapping radiological findings with other serious pathologies require a high degree of clinical suspicion and reliance on histopathological confirmation for accurate diagnosis. In some cases, as we report, only splenectomy can provide a definitive diagnosis and ensure the success of long-term antitubercular therapy.

## Author contribution

Saad Andaloussi: Data collection, analysis and writing the paper.

Saad Annattah: data collection.

Mohammed Eljiar: histopathological data provision and release.

Omar Dalero: conceptualization and supervision.

Zakarya Alami Hassani: clinical data collection and manuscript revision.

Aziz Elmadi: reviewed, supervised and gave recommendations.

## Consent

Written informed consent was obtained from the child's parents for publication of this case report and accompanying images. A copy of the written consent is available for review by the Editor-in-Chief of this journal on request.

## Ethical approval

The Ethical Committee of Mohamed VI University Hospital (CEHUT) does not require IRB approval for reporting individual cases.

Our institution does not require IRB approval for reporting individual cases.

## Guarantor

Saad Andaloussi.

## Funding

This research did not receive any specific grant form funding agencies in the public, commercial, or not-for-profit sectors.

## Registration of research studies

None.

## Declaration of competing interest

Authors declare no conflict of interest related to this case report.
